# Integrin-Linked Kinase Expression Characterizes the Immunosuppressive Tumor Microenvironment in Colorectal Cancer and Regulates PD-L1 Expression and Immune Cell Cytotoxicity

**DOI:** 10.3389/fonc.2022.836005

**Published:** 2022-05-25

**Authors:** Saleh Almasabi, Richard Boyd, Afsar U. Ahmed, Bryan R. G. Williams

**Affiliations:** ^1^ Cancer and Innate Immunity, Centre for Cancer Research, Hudson Institute of Medical Research, Monash University, Clayton, VIC, Australia; ^2^ Cartherics, Hudson Institute of Medical Research, Monash University, Clayton, VIC, Australia; ^3^ Clinical Laboratory Sciences, Applied Medical Sciences, Najran University, Najran, Saudi Arabia

**Keywords:** integrin-linked kinase (ILK), tumor microenvironment (TME), cancer-associated fibroblasts (CAFs), immune cell infiltration, PD-L1, immune evasion, colorectal cancer (CRC)

## Abstract

Integrin-linked kinase (ILK) has been implicated as a molecular driver and mediator in both inflammation and tumorigenesis of the colon. However, a role for ILK in the tumor microenvironment (TME) and immune evasion has not been investigated. Here, we show a correlation of ILK expression with the immunosuppressive TME and cancer prognosis. We also uncover a role for ILK in the regulation of programmed death-ligand 1 (PD-L1) expression and immune cell cytotoxicity. Interrogation of web-based data-mining platforms, showed upregulation of ILK expression in tumors and adjacent-non tumor tissue of colorectal cancer (CRC) associated with poor survival and advanced stages. ILK expression was correlated with cancer-associated fibroblast (CAFs) and immunosuppressive cell infiltration including regulatory T cells (Treg) and M2 macrophages (M2) in addition to their gene markers. ILK expression was also significantly correlated with the expression of different cytokines and chemokines. ILK expression showed pronounced association with different important immune checkpoints including PD-L1. Deletion of the ILK gene in PD-L1 positive CRC cell lines using a doxycycline inducible-CRISPR/Cas9, resulted in suppression of both the basal and IFNγ-induced PD-L1 expression *via* downregulating NF-κB p65. This subsequently sensitized the CRC cells to NK92 immune cell cytotoxicity. These findings suggest that ILK can be used as a biomarker for prognosis and immune cell infiltration in colon cancer. Moreover, ILK could provide a therapeutic target to prevent immune evasion mediated by the expression of PD-L1.

## Introduction

The development of colorectal cancer (CRC) is a multistage process during which mutations in epithelial cells of the intestinal inner layer accumulate. In the early stages of CRC, benign polyps are formed; however, an accumulation of specific mutations in these polyps results in the formation of adenomas which have the potential to develop to a more advanced stage of cancer (1). According to the International Agency for Research on Cancer, in 2018 new cases of CRC were around 1.85 million and resulted in 900,000 deaths in both sexes globally (1). CRC is the third most commonly diagnosed cancer and the second leading cause of cancer deaths worldwide (1). Inflammation as evidenced by inflammatory bowel disease (IBD) is one of the highest risk factors initiating CRC (2). Chronic inflammation is maintained in CRC and other cancers in all stages (3).

The tumor microenvironment (TME) is a region neighboring a tumor that contains diverse cellular components and factors which interact with tumor cells to support tumor growth and metastasis (3). Different components in TME include cancer-associated fibroblasts (CAFs), immune cells, vasculatures, soluble factors, and extracellular matrix (ECM) (4, 5). Immune evasion mediated by immune cells in the TME can be facilitated by tumor associated macrophage (TAM), dendritic cells (DCs), myeloid derived suppressive cells (MDSCs) and regulatory T cells (Treg) promoting anti-tumor immune cell exhaustion and supporting tumor growth (6–11). Infiltrating tumor associated macrophages (TAMs) in the TME, can be polarized into two types that reflect their dual function, M1 which has an anti-tumor function, and M2 which promotes cancer progression (10, 12).

A complex mixture of cytokines and chemokines secreted by cancer cells and CAFs as well as immune cells promote inflammation by recruiting different immune cells into the TME and regulating their functions depending on the context and disease stage (3, 10, 13). In addition, immune checkpoints, regulatory molecules that are either stimulatory or inhibitory, are important to maintain balanced immunity or to avoid autoimmunity. In the cancer context, immune regulatory molecules are dysregulated, and inhibit anti-tumor immune cell function (14, 15). The mechanisms for immune evasion mediated by inhibitory molecules are generally initiated by expressed ligands such as PD-L1 on cancer cells and some immune cells including DCs and TAMs engaging with its receptor PD-1 on T cells which in turn leads to T cell exhaustion and cancer cell escape from immune surveillance (14, 16, 17). The most studied immune inhibitory molecules in cancer immunotherapy are PD-L1 and cytotoxic T lymphocyte-associated antigen-4 (CTLA-4); while the therapeutic targeting of these molecules has demonstrated clinical responses in different cancers in a subset of patients including CRC, further studies are required to fully comprehend immune inhibitory mechanisms in CRC (14, 15).

ILK is an intracellular serine/threonine pseudokinase and adaptor protein localized to focal adhesions and myofilaments that interacts with the β1 integrin cytoplasmic domain (18–20). While this interaction results in the phosphorylation of serine and threonine residues of β1-integrin (18), ILK is defined as a pseudokinase since its kinase-like domain lacks key active sites and likely functions as a non-catalytic signal transducer and adaptor, and mediator protein linking the extracellular matrix with the actin cytoskeleton and downstream signaling pathways (19, 20).

ILK is broadly expressed in many human tissues and cells (21) where it is implicated in the regulation of different cellular processes based on context, including differentiation, proliferation, survival, apoptosis, cell adhesion, angiogenesis, migration and invasion (22). It has been established that ILK overexpression and dysregulation are associated with the development and progression of different cancers including CRC (23–26). Also, its overexpression is associated with a poor survival rate of cancer patients (27–29).

Earlier reports have indicated a function for ILK in TME. For example, ILK has been shown to be involved in endothelial differentiation to CAFs and in DC polarization in response to integrin ligands mediating migration and adhesion (4, 30). In addition, differential expression of CXC chemokines has been shown to be related to ILK (31). Chronic inflammation is well known in cancer promotion (3) and is one of the high-risk factors for developing colon cancer as indicated by IBDs. ILK has been shown to promote inflammation (32–34) and ILK KO in mouse intestinal epithelial cells display a reduction in inflammation of the colon (colitis) and inflammation-induced cancer (colitis-associated cancer) (34, 35). Moreover, we have previously shown that myeloid-ILK deficiency reduced intestinal inflammation in experimental colitis by regulating neutrophil infiltration and cytokine production (32). These studies suggest that while ILK is an essential molecule for promoting the inflammatory tumor microenvironment (36) and its different compartments, its importance in regulating the different TME components remains to be determined.

Here we have investigated expression of ILK in tumors and adjacent non-tumor tissues and correlated ILK expression with several TME factors in solid tumors, particularly in CRC where response to immunotherapy is unresolved (37). We found that ILK is highly expressed in TME and its expression is associated with CAFs and immune cell infiltration. There was also a significant positive correlation between ILK and immunosuppressive cell infiltration and gene markers for TAM/M2 macrophage and Treg cells as well as T cell exhaustion markers. ILK deletion in CRC cell lines reduced NF-κB p65 mediated PD-L1 expression both at the basal level and following IFNγ stimulation, and subsequently enhanced CRC cell sensitivity to NK92 immune cell cytotoxicity *in vitro*. These findings identify a role for ILK in TME and suggest that targeting ILK could be effective for overcoming the immunosuppressive TME in solid tumors like CRC thereby facilitating immunotherapy.

## Materials and Methods

### Immune Cell Infiltration

Evaluation of different immune cell infiltrations as well as CAFs used the Tumor IMmune Estimation Resource (TIMER) platform to determine the correlation between ILK expression and the infiltration of immune cells as well as CAFs in diverse types of cancer from the TGA database (38). CCL13 and IL10 gene expression were examined in term of correlation with immune cell infiltration *via* TIMER platform. Positive correlation indicates that a higher gene expression level is associated with increased immune cell or CAF infiltration and vice versa for negative correlation. Also, the differential expression of ILK mRNA between tumors and their adjacent normal tissues across all TCGA tumors were examined by TIMER. Furthermore, the correlation was evaluated between ILK expression and different gene markers for different immune cell subsets including CD8^+^ and all subsets of T cells, B cells, monocytes, TAMs, M1 and M2 macrophages, neutrophils, NK cells and DCs (39). ILK correlation with markers for diverse functional T cells including T-helper 1 (Th1), T-helper 2 (Th2), follicular helper T (Tfh), T-helper 17 (TH17), Treg and T cell exhaustion was also assessed and the expression of different cytokines and chemokines also assessed for correlation with ILK expression.

The gene markers to define cell types are as follow: CD8A and CD8B for CD8^+^ T cell; CD3D, CD3E and CD2 for all subsets of T cells; CD19 and CD79A for B cell; CD86 and CSF1R for monocyte; CCL2, CD68 and IL10 for TAM; NOS2, IRF5 and PTGS2 for M1; CD163, VSIG4 and MS4A4A for M2; CEACAM8, ITGAM and CCR7 for neutrophil; KIR2DL1, KIR2DL3, KIR2DL4, KIR3DL1, KIR3DL2, KIR3DL3 and KIR2DS4 for NK cell; TBX21, STAT4, STAT1, IFNG and TNF for Th1 cell; GATA3, STAT6, STAT5A and IL13 for Th2 cell; BCL6 and IL21 for Tfh cell; STAT3 and IL17A for TH17; FOXP3, CCR8, STAT5B and TGFB1 for Treg cell; PDCD1, CTLA4, LAG3, HAVCR2 and GZMB for T cell exhaustion; and ACTA2, CXCL12, FAP, PDGFRB, S100A4, TGFB1 and LOX for CAFs.

### ILK Expression in Different Tumor Subsets

ILK mRNA expression levels were examined from different datasets in different cancers compared with normal tissues using the cancer microarray database and web-based data-mining platform Oncomine (40). The thresholds were *P*-value of 0.001, fold change of all, and gene ranking of all. The CRCs datasets were selected and tracked in Oncomine to observe samples used as a normal control for comparison (**Table 1**).

**Table 1:** ILK mRNA expression in Oncomine CRC datasets comparing levels in normal tissues from either adjacent non tumor tissue or tissues from healthy individuals.

Cancer type	*P* value	Fold change	Rank	Sample	Normal tissue source	Ref
**Colon carcinoma**	3.31E-4	1.286	22%	40	Normal colon tissue from healthy individuals	PMID: 20957034
**Colorectal carcinoma**	5.01E-4	1.313	25%	82	Normal colon tissue from healthy individuals	PMID: 20143136
**Colon adenocarcinoma**	3.23E-6	-2.296	2%	36	Adjacent non tumor tissues	PMID: 11306497
**Rectal Mucinous Adenocarcinoma**	3.07E-4	-1.507	12%	28	Paired normal colorectal tissue	TCGA
**Cecum Adenocarcinoma**	1.14E-5	-1.474	22%	44	Paired normal colorectal tissue	TCGA
**Rectal Adenocarcinoma**	3.06E-7	-1.525	24%	82	Paired normal colorectal tissue	TCGA
**Colon Mucinous Adenocarcinoma**	3.53E-4	-1.379	27%	44	Paired normal colorectal tissue	TCGA
**Colon Adenocarcinoma**	1.88E-5	-1.381	31%	123	Paired normal colorectal tissue	TCGA

The dataset GSE95132 was downloaded from the Gene Expression Omnibus (GEO) database that includes 10 CRC patients (10 tumor tissues and their 10 adjacent non-tumor tissues) and 5 non-cancer individuals (5 normal colonic crypts, non-cancer tissues). In this dataset 5 aberrant crypts foci tissues from non-cancer individuals were excluded since these tissues are considered abnormal. ILK mRNA expression (Log2(TPM+1)) was examined in the samples to compare tumors, their adjacent non-tumor tissues and normal tissues. The immune and CAF gene signatures were also examined in the same samples.

GEPIA (Gene Expression Profiling Interactive Analysis) is a web-based tool that provides quick functionalities based on RNA sequencing expression from TCGA and GTEx data (41) and was used to investigate an association between ILK expression, CAFs gene markers and epithelial-mesenchymal transition (EMT) gene markers. Gene markers of different immune cell subsets were also examined using this platform in terms of correlation with ILK expression. In addition, different immune inhibitory gene expression patterns and their correlation with ILK expression were evaluated *via* GEPIA.

The expression of ILK in different cell types was examined *via* the immune cell atlas platform (Single Cell Portal (broadinstitute.org) from a study in colon cancer (42). The cell clusters were presented in t-distributed stochastic neighbor embedding (t-SNE) plots. The cell types examined were epithelial cells including cancer and non-cancer cells (Epi), myeloid cells (Myeloid), plasma cells (Plasma), and T and NK and innate lymphoid cells (TNKILC). Also, FOXP3 and CD163 gene expression was examined here as canonical markers for Tregs and M2 macrophages, respectively.

### ILK Protein Expression

The human protein atlas database https://www.proteinatlas.org/ENSG00000166333-ILK/ pathology was used to investigate the survival probability of colon adenocarcinoma (COAD), stomach adenocarcinoma (STAD), lung squamous cell carcinoma (LUSC) and kidney renal clear cell carcinoma (KIRC) related to ILK protein expression. The COAD patients were divided into low and high ILK mRNA expression groups, below and above the best cut-off (4.57 FPKM, Fragments Per Kilobase of transcript per Million mapped reads) respectively. Data for low ILK expression (n=335) and high ILK expression patients (n=103) were examined. The LUSC patients were divided into low and high ILK mRNA expression groups, below and above the best cut-off (2.16 FPKM) respectively. Low ILK expression patients (n=164) and high ILK expression patients (n=330). The STAD patients were divided into low (n=270) and high (n=84) ILK mRNA expression groups, below and above the best cut-off (3.86 FPKM) respectively. The KIRC patients were divided into low (n=133) and high (n=395) ILK mRNA expression groups, below and above the best cut-off (3.80 FPKM) respectively. For instance, the best expression cut-off refers to the FPKM values that yields greatest survival difference between the two groups.

To further examine the potential clinical role of ILK, the Gene Expression database of Normal and Tumor tissue (GENT2) was used (43) to analyze ILK gene expression and its relationship with the Duke’s stages of 290 CRC patients. Stage A (n=44); stage B (n=94); stage C (n=91); and stage D (n=61).

### Cell Lines

The human CRC cell lines including HCT116, RKO, HT29 and SW480 were obtained from ATCC. The cells were maintained in RPMI-1640 medium with L-glutamine and sodium bicarbonate (Sigma-Aldrich) containing 10% fetal bovine serum (FBS) and Penicillin Streptomycin at 37°C in a 5% humidified CO_2_ incubator. The cells were routinely passaged every 3 days using trypsin. The human NK92 cell line was maintained in RPMI-1640 medium with L-glutamine and sodium bicarbonate containing 20% FBS, Penicillin Streptomycin and 200 IU/mL IL-2 (Miltenyi Biotec). Interferon gamma (IFNγ, Lonza Australia Pty Ltd) was used to stimulate PD-L1 expression in the CRC cell lines.

### Doxycycline Inducible-CRISPR/Cas9 Deletion of ILK

The CRC cell lines were transfected with a doxycycline inducible-CRISPR/Cas9 system (44), which was designed to delete the ILK gene. Multiple single cell clones were derived, cryopreserved and the efficacy of CRISPR/Cas9-driven ILK deletion in these cells was verified by Western blots. Aliquots of frozen cells were thawed and grown for 4 days. After trypsinization, 100 x10^3^ cells/well were seeded into a 6-well plate and duplicated into 2 sub-populations. The plates were incubated for cell adherence overnight at 37°C in 5% CO_2_ and the following day, one sub-population per cell line treated with 2 µg/ml doxycycline to induce CRISPR/Cas9 to delete the ILK gene. The cells were cultured for 3 days, the doxycycline washed from the cells and cells sub-cultured. The doxycycline-treated cells were then designated as ILK knockdown (KD) or +Dox while the non-treated cells were designated as WT or -Dox.

### Western Blotting

After seeding and reaching near confluence, cells were either left untreated or treated depending on the experiment. Total proteins were extracted using RIPA lysis buffer supplemented with protease and phosphatase inhibitors (MERK and Roche, respectively). After denaturation with 3x sample buffer, the proteins were electrophoresed in 12% SDS- polyacrylamide gels (Bio-Rad) and transferred to polyvinylidene difluoride membranes (Millipore). The membranes were incubated with primary rabbit polyclonal Ab against ILK (Cell Signaling Technology #3856), NF-κB p65 (Cell Signaling Technology #8242), Phospho-NF-κB p65 Ser536 (Cell Signaling Technology #3033) or PD-L1 (Cell Signaling Technology #13684). Protein loading in each well was verified by the detection of actin using a pan-actin mouse polyclonal Ab (NeoMarker. The membranes were incubated with secondary anti-rabbit Alexa Fluor 680– or anti-mouse Alexa Fluor 760-conjugated Ab (Invitrogen). Detection was visualized using Odyssey CLX (Licor). The densitometry of the bands was quantitated by ImageStudioLite.

### Cytotoxicity Assay by Co-Culturing NK92 Immune Cells With CRC Cells

Cytotoxicity activity of NK92 immune cells against -Dox and +Dox CRC cells was examined by co-culturing and subsequent assessment of cell density destruction visualized by crystal violet staining. At zero time point, optimal -Dox and +Dox CRC cell number (target) was seeded into a 96-well plate. The optimal cell number for HCT116 and SW480 is 25 x 10^3^ cells/well, and for RKO and HT29 is 50 x 10^3^ cells/well. The following day after CRC cells adhesion the NK92 immune cells (effector) were added over the target cells at different effector to target (E:T) ratios ranging between 1:4 to 4:1. The effector cells were incubated for either 24hr or 48hr depending on CRC cell line. The control cells (T, target only) were not incubated with effector cells. Next, effector cells and any dead target cells were washed off by gentle media change. Remaining adherent cells were incubated for 24hr to 48hr until control cell density reached 100%. The cells next were washed and fixed with 100% cold methanol for 20 mins and stained with 0.5% crystal violet for 5 mins to visualize cell density. The plates were imaged and cell density destruction by effector cells quantitated by ImageJ software. The cell density was normalized to control cells.

### Statistical Analysis

Survival curves were generated *via* the human protein atlas and Kaplan–Meier curve. The data generated in Oncomine are displayed as *P*-values, fold change, and gene rank. The correlation between gene expression was evaluated by Spearman’s correlation (R) and statistical significance as indicated by *P*-value. The gene expression correlation with immune cell and CAFs infiltration is considered significant at *P*-value <0.05, and the infiltration level is categorized based on R values as follow: ± 1 to ±0.28 is strong, ± 0.27 to ±0.19 is moderate, ± 0.18 to ±0.10 is weak and ±0.09 to 0 is no correlation. The difference between cancer types in term of ILK correlation with genes expression were plotted as R values in heatmaps. The results were analyzed using GraphPad Prism8. Unpaired t-tests were used to analyze the difference between two groups and Ordinary One-way Anova with multiple comparisons was used to analyze the difference between more than two groups. The data were displayed as mean ± SEM. The significant *P*-value is <0.05. *P < 0.05, **P < 0.01, ***P < 0.001.

## Results

### ILK Expression in Cancers and Its Implication in Prognosis

#### Differential Expression of ILK in Different Cancers

**Figure 1:**
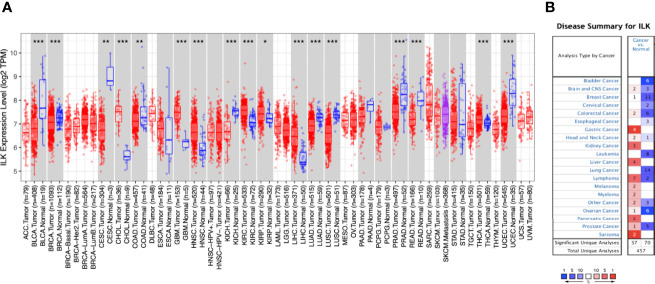
Differential expression of ILK mRNA in cancers and normal tissues. **(A)** Differential ILK mRNA expression between different human cancers and their adjacent normal tissues from TCGA was evaluated *via* TIMER (*P < 0.05, **P < 0.01, ***P < 0.001). **(B)** The expression levels of ILK mRNA in independent datasets in different cancers compared with normal tissues were assessed by Oncomine. The thresholds were *P*-value of 0.001, fold change of all, and gene ranking of all. The red cells represent cancer datasets and the blue cells normal datasets, and the numbers inside the cells indicate the number of independent datasets. The color intensity is an indicator of the gene rank percentile.

The expression of ILK in different cancers compared with their adjacent normal tissues from the TCGA was determined using the TIMER database. The results (**Figure 1A**) showed that ILK expression is significantly higher in CHOL, GBM, HNSC, KIRC, KIRP, LIHC, THCA compared with their adjacent normal tissues. On the other hand, ILK is significantly lower in BLCA, BRCA, CESC, COAD, KICH, LUAD, LUSC, PRAD, READ and UCEC compared with their adjacent normal tissues (**Figure 1A**). These results indicate that ILK levels vary in different cancers compared with their adjacent normal tissues.

It has been established previously that ILK is upregulated in different cancers compared to normal tissues (22, 45). However, as shown above some cancers exhibit the opposite. To investigate this further we examined the expression level of ILK mRNA from different datasets in different cancers compared with normal tissues using the Oncomine platform. Overall, ILK mRNA expression is higher in certain cancers compared with normal tissues in 37 datasets, whereas its expression is lower in other cancers compared with normal tissues in 70 datasets (**Figure 1B**). For instance, ILK expression is higher in brain, breast, colorectal, gastric, head and neck, kidney, liver, lymphoma, melanoma, myeloma, ovarian, pancreatic, prostate, sarcoma cancers in some datasets (**Figure 1B**). In contrast, in different datasets lower expression of ILK is observed in bladder, brain, breast, cervical, colorectal, esophageal, head and neck, leukemia, lung, lymphoma, ovarian and prostate cancers (**Figure 1B**). Therefore, different independent datasets exhibit contradictory results for ILK expression levels in cancers and normal tissues.

To investigate the possible reason behind such contradictory results from different datasets, CRCs were selected and tracked in Oncomine to determine which samples have been used as normal controls for comparison as it has been recognized that adjacent non-tumor tissues may exhibit abnormal genetic profiles (46–48). We focused on solid tumors, particularly on CRC and two datasets that display higher ILK mRNA expression in CRC compared with normal tissue used normal colon tissues from healthy individuals. In contrast, 6 datasets that display lower ILK mRNA expression in CRC compared with normal tissue and used adjacent non-tumor tissues from cancer patients (**Table 1**). Therefore, these results indicate that ILK expression is upregulated in adjacent non-tumor tissues from CRC patients and constitute a component of the TME.

#### Upregulation of ILK Expression in CRC Tumors and Its Correlation With Prognosis

COAD, LUSC, KIRC and STAD were selected for further investigation since COAD and LUSC showed significantly lower ILK expression in tumors compared with their adjacent normal tissues, while KIRC showed significantly higher expression in tumors compared with adjacent normal tissues, and STAD showed intermediate expression of ILK in tumors compared with adjacent normal tissues (**Figure 1A**). Nonetheless, the focus will be on COAD tumors as a solid tumor model.

**Figure 2:**
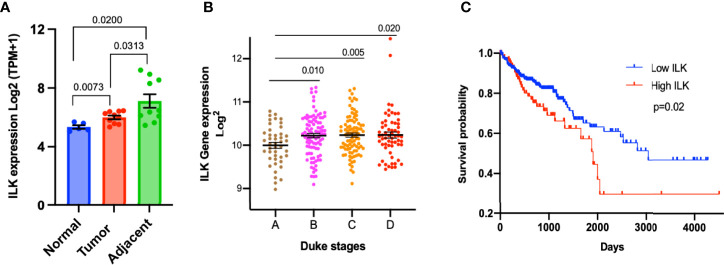
Upregulation of ILK expression in CRC tumors and its correlation with prognosis. **(A)** The ILK expression levels (Log2(TPM+1)) were examined in a dataset (GSE95132) includes 10 CRC patients (10 CRC tumor tissues (Tumor) and their 10 adjacent non-tumor tissues (Adjacent)) and 5 non-cancer individuals (5 normal colonic crypts (Normal)). The dataset GSE95132 was downloaded from GEO database. **(B)** ILK expression and its relationship with the Duke’s stages of CRC compared by using the GENT2 database. The stages pf CRC (n = 290) are stage A (n = 44); stage B (n = 94); stage C (n = 91); and stage D (n = 61). The figure was reproduced from the Gene Expression database of Normal and Tumor tissue (GENT2) at http://gent2.appex.kr/gent2/. **(C)** Kaplan–Meier curve of overall survival rate between COAD patients showing high and low ILK mRNA expression in the TCGA database through the human protein atlas. The patients were divided into low and high ILK mRNA expression groups, below and above the best cut-off (4.57 FPKM) respectively. Low ILK expression patients (n = 335) and high ILK expression patients (n = 103). The figure was reproduced from the human protein atlas at https://www.proteinatlas.org/ENSG00000166333-ILK/pathology/colorectal+cancer/COAD. Error bars are represented as mean ± SEM. *P*-value was analyzed with an unpaired t-test. Significant *P*-value is <0.05. The significant *P*-value is <0.05.

In order to confirm the upregulation of ILK in adjacent non-tumor tissues and to determine the overall ILK expression level in cancer tissues, we examined the differential expression of ILK in colon tissues from CRC patients and non-cancer individuals in the dataset GSE95132 downloaded from GEO. The results (**Figure 2A**) showed that ILK expression is significantly higher in primary tumors and their adjacent non-tumor tissues from CRC patients compared with individual’s non-cancer tissues (**Figure 2A**). Also, ILK expression is significantly higher in adjacent non-tumor tissues compared with primary tumors from the same patients (**Figure 2A**). This result is in line with the result of ILK differential expression in the COAD presented in **Figure 1A**. Thus, these results are not only consistent with previous observations reporting upregulation of ILK in cancer tissue but also suggest that ILK may be implicated in the TME as it is upregulated in both primary tumors and surrounding tissues.

In order to examine the association of ILK expression with stages of colon cancer, the data from the GENT2 platform were utilized and gene expression of ILK investigated in different Duke’s stages of 290 colon cancer patients. The results showed that ILK expression is upregulated in advanced Duke’s stages B to D compared with stage A, suggesting a role in cancer invasion and metastasis (**Figure 2B**).

To further investigate the role of ILK in prognosis, we used the TCGA database through the human protein atlas to analyze the ILK expression and its relationship with the overall survival of 438 COAD patients. The results showed that the overall survival rate is significantly lower in COAD patients showing high ILK mRNA expression levels compared to those with low ILK expression (**Figure 2C**). Also, the high ILK expression is observed to be associated with poor survival rate in STAD and LUSC (**Supplementary Figure 1**). Collectively, these results indicate a potential role for ILK in colon cancer prognosis.

### ILK Expression in the TME

#### ILK Expression Is Positively Correlated With CAFs Infiltration and EMT Markers

**Figure 3:**
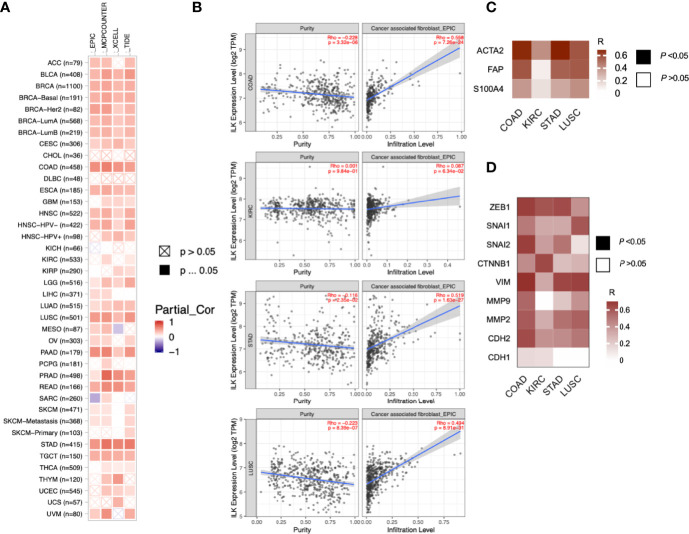
ILK is correlated with CAF infiltration and EMT markers. **(A)** ILK expression is associated with CAF infiltration in most cancers as analyzed using TIMER. EPIC, MCPCOUNTER, XCELL and TIDE as shown in the heat map are different methods for cell quantification based on gene expression. Since most fibroblasts and immune cells are negatively correlated with tumor purity, the correlation is adjusted by purity (referred as Partial_Cor) partial spearman’s correlation rho. **(B)** The ILK expression is correlated with CAFs infiltration in COAD, KIRC, STAD and LUSC *via* TIMER. **(C)** Heat map showing the correlation in expression between ILK and key gene markers for CAFs in COAD, KIRC, STAD and LUSC tumors *via* GEPIA. **(D)** Heat map showing the correlation in expression between ILK and different gene markers for EMT in COAD, KIRC, STAD and LUSC tumors *via* GEPIA. The values in the heat map are for Spearman’s correlation Rho, R. The scale on the right of the heat map is for R values. All colored squares are statistically significant whereas white squares are not statistically significant. The R values above 0.08 are statistically significant. Significant *P*-value is **<**0.05.

As the previous data showed ILK upregulation in tissues surrounding tumors of CRC patients, we asked whether ILK expression is increased in different TME cellular components including infiltrating CAFs and immune cells. CAFs are one of the most abundant stromal components of the TME (5). Moreover, it has been suggested that ILK is involved in fibroblast survival and CAF differentiation (4, 49). Accordingly, we investigated ILK association with CAFs in the TME across different cancers *via* the TIMER platform. The results (**Figure 3A**) showed that ILK expression is significantly associated with CAFs infiltration in most cancers. More specifically, the association with CAFs was significantly higher in COAD, LUSC and STAD, whereas it is low in KIRC (**Figure 3B**).

To confirm this, we also explored, *via* GEPIA, the associations between ILK expression and CAF gene markers and related genes including ACTA2, CXCL12, FAP, PDGFRB, S100A4, TGFB1 and LOX. It was found that almost all of the markers were significantly correlated with ILK expression in COAD, STAD and LUSC compared with lower correlation in KIRC, the R averages being 0.60, 0.54, 0.47 and 0.28 respectively (**Table 2**). The key gene markers for CAFs presented in a heatmap show a clear correlation with ILK expression in COAD, STAD and LUSC, but a weaker correlation in KIRC (**Figure 3C**). Since CAFs are involved in EMT induction and facilitate invasion and metastasis (50) the correlation between ILK and gene markers of EMT were also examined using GEPIA. The results showed that ILK displayed a positive significant correlation with EMT markers in COAD including ZEB1, SNAI1, SNAI2, CTNNB1, VIM, MMP9, MMP2 and CDH2 compared with weaker correlation with CDH1 epithelial marker (**Figure 3D**). Also, STAD and LUSC displayed positive correlation between ILK and EMT markers, whereas the correlation was weaker in KIRC (**Figure 3D**). Taken together, the results suggest that ILK expression is correlated with the infiltration of CAFs and their markers and EMT markers in different cancers.

**Table 2:** ILK correlation with CAF gene markers.

*Gene markers*	COAD		KIRC		STAD		LUSC	
	R	*P*	R	*P*	R	*P*	R	*P*
***ACTA2***	0.68	***	0.41	***	0.7	***	0.57	***
***CXCL12***	0.6	***	0.44	***	0.5	***	0.39	***
***FAP***	0.57	***	0.1	*	0.54	***	0.56	***
***PDGFRB***	0.66	***	0.51	***	0.61	***	0.6	***
***S100A4***	0.38	***	0.15	**	0.33	***	0.41	***
***TGFB1***	0.63	***	0.28	***	0.58	***	0.35	***
***LOX***	0.66	***	0.09	*	0.53	***	0.43	***

GEPIA based correlation of ILK expression with CAF gene markers in tumor tissues from TCGA. R is Spearman’s correlation Rho value. Significant P-value is <0.05. *P < 0.05, **P < 0.01, ***P < 0.001.

#### ILK Expression Is Negatively Correlated With Tumor Purity and Positively Correlated With Immune Cell Infiltration in COAD, STAD and LUSC

**Figure 4:**
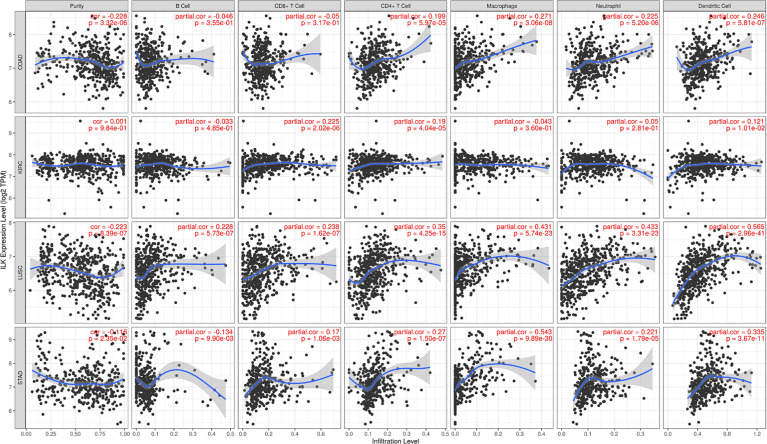
ILK expression is negatively correlated with tumor purity and positively correlated with immune cell infiltration in COAD, STAD and LUSC. The correlations between ILK gene expression and tumor purity are on the left panel. The correlation between ILK and immune cells including B cells, CD8^+^ T cells, CD4^+^ T cells, macrophages, neutrophils and DCs are corrected by tumor purity (partial.cor, partial Spearman’s correlation rho). (upper row) COAD; (second row) KIRC; (third row) LUSC; (lower row) STAD. Significant *P*-value is < 0.05.

We have previously shown that ILK expression in myeloid cells is involved in regulating neutrophil infiltration in experimental colitis in mice (32). This suggests that ILK could have a role in the infiltration of different immune cells in the TME in a cancer context. To examine this, 39 cancers were assessed using the TIMER platform to investigate the correlation between ILK expression and tumor purity as well as infiltration of B cells, CD8^+^ T cells, CD4^+^ T cells, macrophages, neutrophils and DCs. Genes highly expressed in the TME are expected to have negative associations against tumor purity, while the opposite is expected for genes highly expressed in the tumor cells (38). The results overall show that ILK has significant negative correlations with tumor purity in 12 cancers (**Supplementary Figure 2**). The negative correlations between ILK and tumor purity exhibited positive correlations with immune cell infiltration. Furthermore, ILK was significantly correlated with B cells and CD8^+^ T cells in 10 cancers, with CD4^+^ T cells in 24 cancers, with macrophages and neutrophils in 23 cancers, and with DCs in 28 cancers (**Supplementary Figure 2**). The correlation values were overall higher in innate immune cells including DCs, macrophages and neutrophils. More specifically, it was found that ILK expression is negatively correlated with tumor purity in COAD, LUSC and STAD which in turn reflected significant positive associations with infiltration of CD4^+^ T cells, macrophages, neutrophils and DCs (**Figure 4**). In contrast, ILK expression in KIRC is not negatively correlated with tumor purity and showed weaker associations with immune cell infiltration compared with the other 3 cancer types (**Figure 4**). Therefore, ILK expression is negatively correlated with tumor purity in COAD, LUSC and STAD indicating an upregulation in the TME and positive correlation with immune cell infiltration levels.

#### ILK Expression in Colon Cancer TME Cell Types

**Figure 5:**
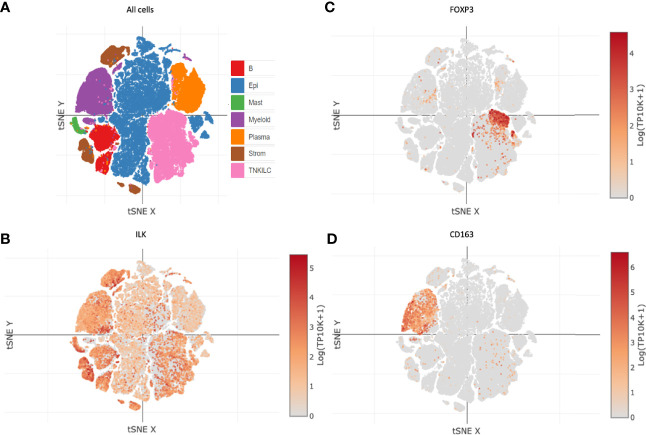
ILK expression in different cell types in colon cancer. ILK expression was examined in patient colon cancers *via* the immune cell atlas platform [Single Cell Portal (broadinstitute.org)]. **(A)** t-distributed stochastic neighbor embedding (t-SNE) plot showing B cells **(B)**, epithelial cancer and non-cancer cells (Epi), myeloid cells (Myeloid), plasma cells (Plasma), and T and NK and innate lymphoid cells (TNKILC) of colon cancer. **(B)** ILK expression in all cell clusters. **(C)** FOXP3 expression for Tregs in all cell clusters. **(D)** CD163 expression for M2 macrophages in all cell clusters.

Since there is a clear ILK association with infiltrating CAFs and immune cells as described above, we also determined whether ILK was expressed in different cell types. Distribution of different cell types including cancer and non-cancer cells from colon cancer patients were investigated in a tSNE plot *via* the immune cell atlas platform (**Figure 5A**). Examination of ILK gene expression revealed that all seven cell lineages express ILK (**Figure 5B**). For instance, ILK expression was observed in cell clusters for epithelial cells, myeloid cells, TNKILC, stromal cells, B cells, mast cells and plasma cells (**Figure 5B**). In addition, while ILK expression levels are variable among cell types, high expression levels are apparent in stromal cells, myeloid cells, B cells, TNKILC and epithelial cells relatively to plasma cells and mast cells (**Figure 5B**). Moreover, examination of FOXP3 and CD163 gene markers for Treg and M2 cells respectively, key immunosuppressive cells in the TME revealed that both genes are expressed in two distinct cell clusters coincident with ILK expression (**Figures 5B–D**). Accordingly, these results suggest that ILK is expressed in most cells in the TME including epithelial cancer, immune and stromal cells, but there is also indicate a concordance between ILK expression and immunosuppressive cells.

#### ILK Expression Is Positively Correlated With Infiltrating Immunosuppressive Cells and Their Gene Markers in COAD, STAD and LUSC

**Figure 6:**
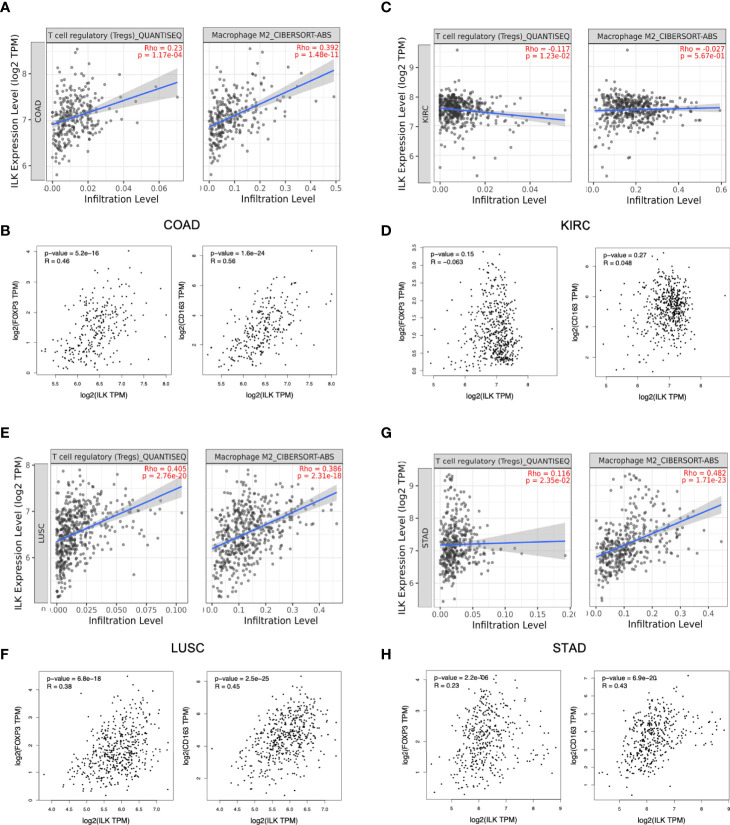
ILK expression is correlated with Tregs and M2 macrophages infiltration and their gene markers in COAD, LUSC and STAD. **(A, C, E, G)** ILK expression correlation with Tregs and M2 macrophages infiltration in COAD, KIRC, LUSC and STAD *via* TIMER platform. **(B, D, F, H)** Gene markers for M2 macrophage (CD163), Treg cells (FOXP3) and their correlation with ILK in COAD, KIRC, STAD and LUSC *via* GEPIA. QUANTISEQ and CIBERSORT-ABB are different methods used *via* TIMER for cell quantification based on gene expression. Since most immune cells are negatively correlated with tumor purity, the correlation is adjusted by purity partial spearman’s correlation rho. R is Spearman’s correlation Rho value. The significant *P*-value is <0.05.

While the above results showed a potential role for ILK in infiltrating immune cells, the implication for cancer is unclear. To attempt to answer this question, the correlation of ILK expression with infiltrating Tregs and M2 macrophages was investigated *via* TIMER as key immunosuppressive immune cells in the TME. The results showed that ILK is significantly correlated with both infiltrating immunosuppressive cells in COAD, LUSC and STAD but not in KIRC (**Figures 6A, C, E, G**). To confirm the association with the mentioned immunosuppressive cells infiltration, FOXP3 marker for Treg and CD163 marker for M2 macrophage were also examined *via* GEPIA. The results displayed that ILK significantly correlated with both genes in COAD, LUSC and STAD but not in KIRC (**Figures 6B, D, F, H**). Thus, the results showed that ILK expression is potentially associated with the infiltrating immunosuppressive cells as well as their markers and that is possibly tumor specific.

Furthermore, several gene markers for functional immune cells and immunosuppressive cells were evaluated *via* TIMER platform as shown in a previous study (39). Interestingly, the results showed that ILK is positively correlated with gene markers for monocytes, TAM, M2 macrophages, neutrophils and DCs in COAD, STAD and LUSC (**Supplementary Table 1**). Also, ILK is positively correlated with Treg and T cell exhaustion markers in COAD, STAD and LUSC (**Supplementary Table 1**). Less association levels of ILK expression were noticed with the above immune cells’ gene markers in KIRC.

To confirm the above results from TIMER analysis, the gene markers for monocytes, TAM, M2 macrophages, neutrophils and DCs as well as Treg and T cell exhaustion in COAD, KIRC, STAD and LUSC were also examined by using the GEPIA platform. The results showed that almost all markers of these immune cell subsets showed significant positive correlations with ILK expression and that is consistent with the data from TIMER (**Table 3**). The R averages are 0.48, 0.34, 0.36 and 0.17 in COAD, STAD, LUSC and KIRC respectively.

**Table 3:** ILK expression is positively correlated with the expression of immune cell gene markers.

	*Gene markers*	COAD		KIRC		STAD		LUSC	
		R	*P*	R	*P*	R	*P*	R	*P*
***Monocyte***	***CD86***	0.56	***	0.11	**	0.37	***	0.47	***
	***CSF1R***	0.59	***	0.27	***	0.51	***	0.53	***
***TAM***	***CCL2***	0.59	***	0.36	***	0.43	***	0.49	***
	***CD68***	0.51	***	0.1	*	0.28	***	0.44	***
	***IL10***	0.52	***	0.15	**	0.41	***	0.43	***
***M2 Macrophage***	***CD163***	0.56	***	0.04	0.27	0.43	***	0.45	***
*** ***	***VSIG4***	0.6	***	0.12	*	0.52	***	0.47	***
*** ***	***MS4A4A***	0.57	***	0.15	**	0.48	***	0.46	***
***Dendritic cell***	***HLA-DPB1***	0.5	***	0.24	***	0.31	***	0.48	***
*** ***	***HLA-DQB1***	0.23	***	0.15	**	0.1	*	0.3	***
*** ***	***HLA-DRA***	0.41	***	0.19	***	0.25	***	0.47	***
*** ***	***HLA-DPA1***	0.47	***	0.21	***	0.29	***	0.48	***
	***CD1C***	0.43	***	0.33	***	0.36	***	0.28	***
*** ***	***NRP1***	0.66	***	0.59	***	0.55	***	0.51	***
*** ***	***ITGAX***	0.54	***	0.02	0.52	0.37	***	0.35	***
***Neutrophils***	***CEACAM8***	-0.07	0.21	0.08	0.055	0	0.94	0.14	*
*** ***	***ITGAM***	0.61	***	0.22	***	0.43	***	0.44	***
	***CCR7***	0.4	***	0.14	*	0.33	***	0.26	***
***Treg***	***FOXP3***	0.46	***	-0.06	0.15	0.23	***	0.38	***
	***CCR8***	0.48	***	0.02	0.65	0.36	***	0.38	***
*** ***	***STAT5B***	0.34	***	0.62	***	0.55	***	0.07	0.12
*** ***	***TGFB1***	0.63	***	0.28	***	0.58	***	0.35	***
***T cell exhaustion***	***PDCD1***	0.34	***	-0.02	0.65	0.24	***	0.22	***
*** ***	***CTLA4***	0.39	***	0.03	0.42	0.16	**	0.28	***
*** ***	***LAG3***	0.29	***	-0.04	0.32	0.16	*	0.12	*
*** ***	***HAVCR2***	0.59	***	0.13	*	0.42	***	0.46	***
	***GZMB***	0.18	*	0.16	**	0.1	*	0.12	*

Correlations of ILK with gene markers expression in tumor tissues from TCGA analyzed via GEPIA. R is Spearman’s correlation Rho value. The significant P-value is <0.05. *P < 0.05, **P < 0.01, ***P < 0.001.

For instance, ILK was significantly associated with gene markers for TAM and M2 like CCL2, CD68, IL10, CD163, VSIG4 and MS4A4A. Also, ILK is significantly associated with several markers for DCs such as ITGAX, NRP1, HLA-DPB1 and others. In addition, ILK is associated with neutrophils markers like ITGAM and CCR7. The functional T cell markers also showed association with ILK expression, particularly, FOXP3 and TGFB1 for Treg and PDCD1 (PD-1), CTLA-4, LAG3 and HAVCR2 (TIM-3) for T cell exhaustion. The indicated markers overall showed a stronger association with ILK in COAD, STAD and LUSC compared with KIRC. Therefore, the results indicate that ILK may be involved in immunosuppressive cell infiltration and regulation of functional immune cells like polarization of macrophages and T cell exhaustion.

Furthermore, the above immune as well as CAF gene signatures were investigated in the same samples (dataset GSE95132) presented in the **Figure 2A**. We found that ILK, immune and CAF gene markers overall are upregulated in the primary tumors and their adjacent non-tumor tissues compared with non- cancer tissues (**Supplementary Figures 4A, B**). ILK expression was variable among patients, and ILK and the other genes exhibit a similar expression trend. In contrast the normal tissues from non-cancer individuals exhibit low expression of ILK and the other genes.

#### ILK Expression Is Significantly Correlated With Different Cytokines and Chemokines Gene Expression in COAD, STAD and LUSC, and This Correlation Potentially Predicts the Infiltrating Immunosuppressive Cells

**Figure 7:**
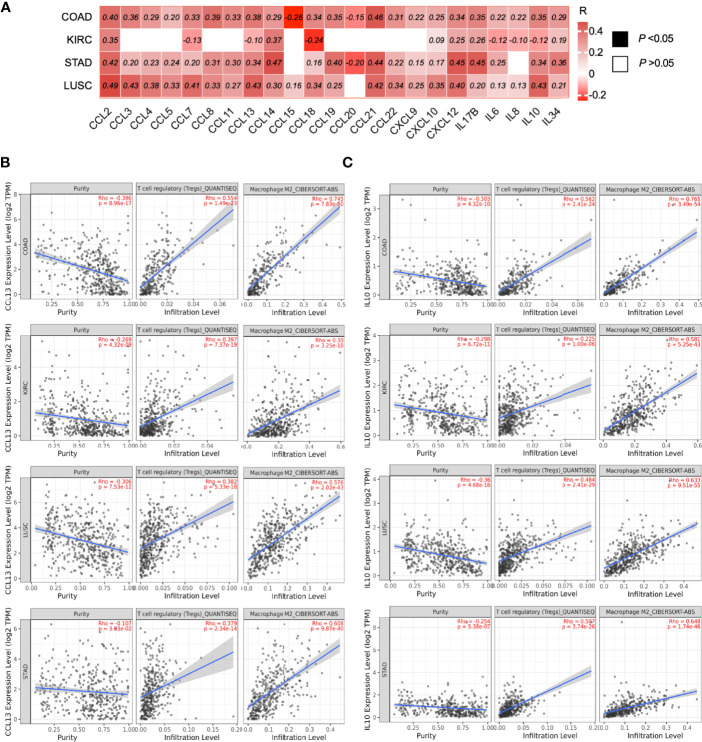
ILK expression is correlated with the expression of chemokines and cytokines implicated in immunosuppressive cell infiltration. **(A)** Heat map of different chemokine and cytokine gene expression and their correlations with ILK in COAD, KIRC, STAD and LUSC were examined *via* TIMER. The values in the heat map are for the spearman’s correlation Rho, R. The scale on the right of the heat map is for R values. All colored squares are statistically significant whereas white squares are not statistically significant. The R values above 0.08 are statistically significant. **(B)** CCL13 correlation with tumor purity, Treg and M2 infiltration. **(C)** IL10 correlation with tumor purity, Treg and M2 infiltration. Significant *P*-value is <0.05.

As the previous data suggested that ILK has an impact in infiltration and regulation of immune cells, we asked whether ILK could contribute to chemokine and cytokine secretion in the TME, as these secretions have an implication in recruiting and regulating immune cell functions as well as cancer progression (3, 10, 13). The expression of several cytokines and chemokines were assessed by TIMER analysis to investigate their correlation with ILK expression in COAD, KIRC, STAD and LUSC. The results showed that ILK has significant correlations with most cytokines and chemokines in COAD, STAD and LUSC compared with KIRC (**Figure 7A**). The highest positively correlated genes in COAD were CCL2, CCL8, CCL13, CCL19, CCL21, IL17B, IL10.

In addition, ILK expression has a significant positive correlation with CCL13 and IL10 expression in COAD, STAD and LUSC; in contrast there is a negative correlation in KIRC (**Figure 7A**). Both CCL13 and IL10 gene expression were examined *via* TIMER in terms of their correlation with Treg and M2 macrophage infiltration. The results showed that both CCL13 and IL10 expression are significantly associated with the infiltrating immunosuppressive cells in all four cancers, including KIRC (**Figures 7B, C**). These results indicate that ILK expression is tightly correlated with the gene expressions for cytokines and chemokines in different tumor contexts, and it is suggested that ILK plays a role in the TME immune cell functions and infiltrations *via* its link with cytokines and chemokines.

#### ILK Expression Is Significantly Correlated With Immune Inhibitory Genes in COAD

**Figure 8:**
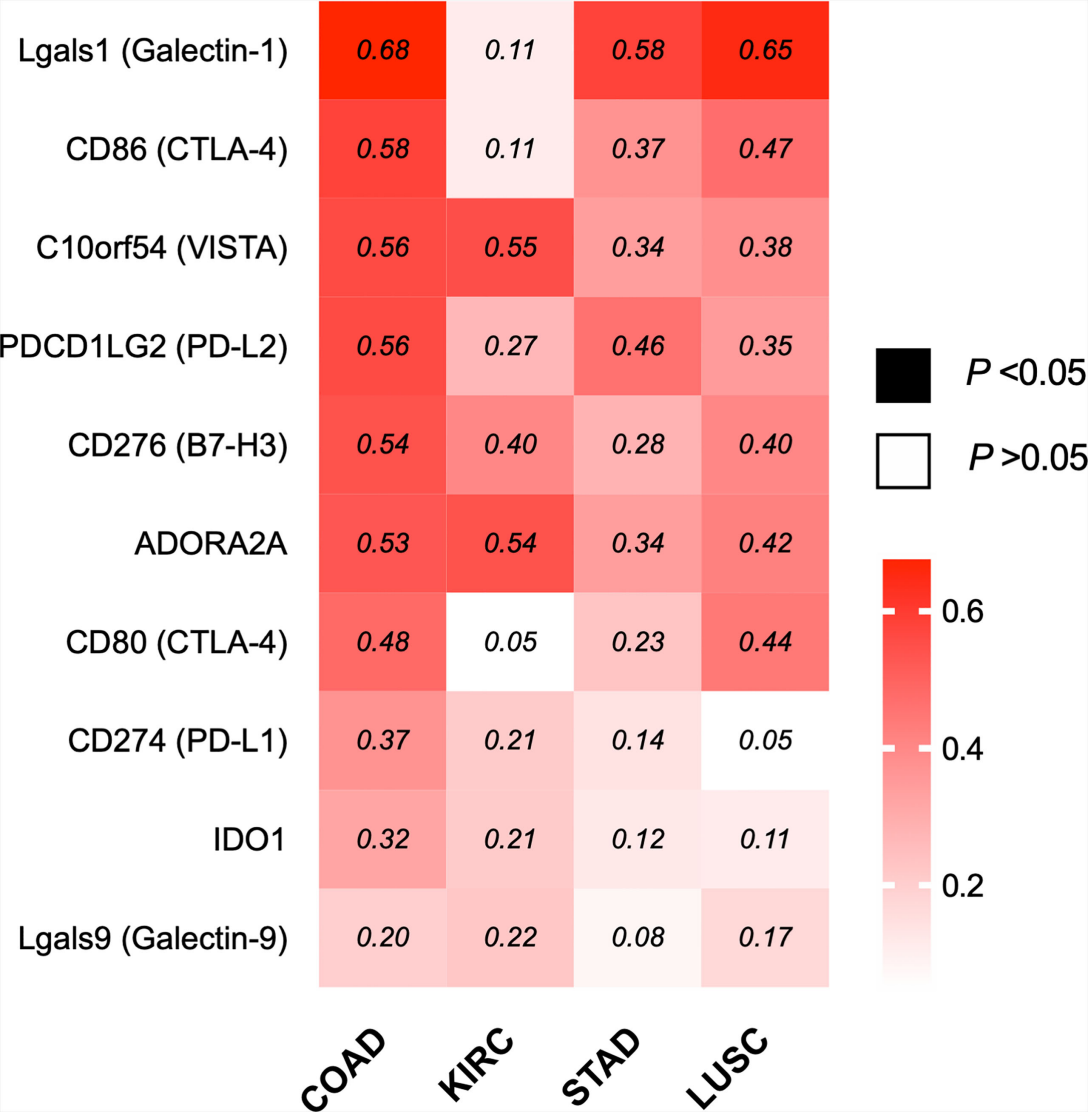
ILK expression is significantly correlated with several immune inhibitory genes in COAD. Heat map of different immune inhibitory genes and their correlation with ILK in COAD, KIRC, STAD and LUSC *via* GEPIA. The values in the heat map are Spearman’s Rho values (R). The scale on the right of the heat map is for R values. The colored squares are statistically significant while the white squares are not statistically significant. The R values that are above 0.08 are statistically significant. Significant *P*-value is <0.05.

Based on the correlation of ILK with the different components in TME particularly immunosuppressive cell infiltration and their markers, it remains possible that ILK could also have a function in regulating immune evasion *via* immune checkpoint regulation, one of the core mechanisms the TME employs to suppress antitumor immunity (15, 51). Accordingly, different inhibitory molecules were evaluated by GEPIA in terms of correlation with ILK, including Lgals1, Lgals9, CD274, PDCD1LG2, CD80, CD86, C10orf54, ADORA2A, IDO1 and CD276. The results (**Figure 8**) showed that ILK expression has a positive significant correlation with the immune inhibitory molecules with a more marked correlation in COAD, LUSC and STAD respectively compared with weaker correlation in KIRC. Therefore, these results suggest that ILK has the potential to impact on regulating immune checkpoints expression, particularly in COAD.

### ILK Is Involved in PD-L1 Regulation in CRC Cell Lines

#### ILK KD Reduced Basal PD-L1 Expression in RKO CRC Cells *via* NF-κB p65 Regulation

Since ILK correlation with the immune inhibitory molecules was greater in COAD (**Figure 8**), we decided to further investigate whether ILK is implicated in regulating PD-L1 (CD274) protein expression in CRC cell lines *in vitro*, our focused cancer model in this study. PD-L1 was selected and examined here because this is in advanced phase of clinical trials and that need more optimization and combining therapy to solve issues of its limitations (52). While blocking PD-L1 has shown promising results in cancer therapy, not all patients respond, and some develop resistance (37, 53). The microsatellite instable (MSI) tumors showed better response than microsatellite stable (MSS) tumors to PD-L1 inhibitors (37, 53, 54). We thought that examining PD-L1 expression in MSI and MSS CRC cell lines might be important in presence and absence of ILK to provide useful information for future therapeutic implications. Examples of MSI cell lines are HCT116 and RKO whereas both HT29 and SW480 are recognized within the MSS lines (55).

**Figure 9:**
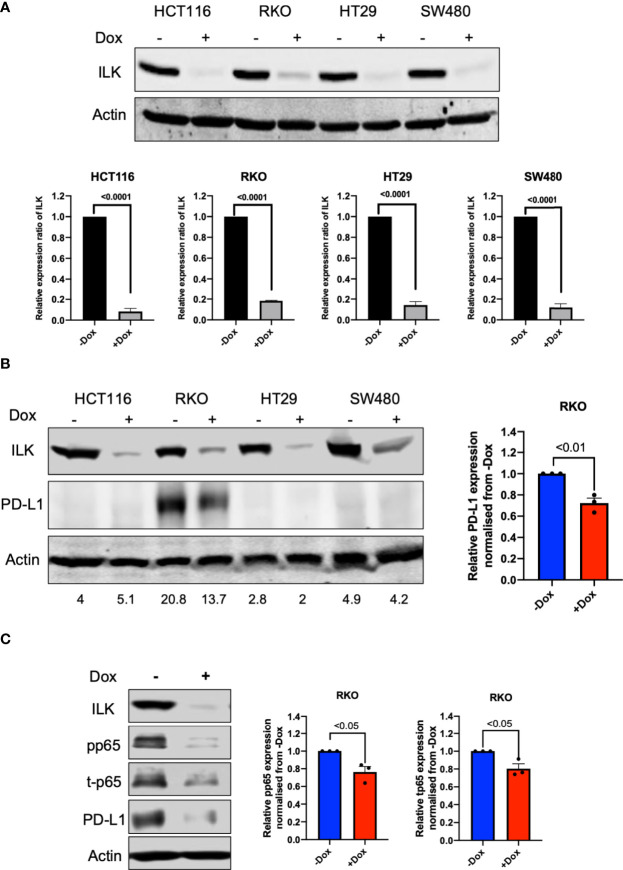
ILK KD reduced basal PD-L1 expression in RKO CRC cells *via* NF-κB p65 regulation. [**(A)**, upper] Western blot showing expression of ILK protein. [**(A)**, lower] Quantitation of ILK protein, the means were normalized to -Dox (n = 3). Cells were seeded into a 6-well culture plate and duplicated into 2 sub-populations and incubated overnight. One sub-population per cell line was treated with 2 µg/ml doxycycline to induce CRISPR/Cas9 to delete the ILK gene over 3 days. The doxycycline was washed from the cells and the cells sub-cultured for growing. The cells were then harvested for testing ILK protein expression *via* western blot and the Dox-treated cells are verified as ILK KD cells. **(B)** The western blot of PD-L1 protein expression in CRC cell lines at the basal level. **(C)** ILK KD reduced NF-κB p65/PD-L1 protein expression in the RKO CRC cell line. (left) The western blot showed the effect of ILK KD in RKO cells on PD-L1 and NF-κB p65 (total and phosphorylated) protein expression at 4 hr serum stimulation following overnight starvation. (right) The quantitation of PD-L1, p65 phosphorylation (pp65) and p65 total protein expression (t-p65). Quantitation of protein expression normalized to -Dox. Actin was used as an internal control. Error bars are represented as mean ± SEM (n = 3). *P*-value was analyzed with an unpaired t-test. The significant *P*-value is < 0.05.

To determine a role for ILK in PD-L1 regulation in the epithelial CRC cell lines, doxycycline inducible-CRISPR/Cas9 was utilized to delete the ILK gene in HCT116, RKO, HT29 and SW480 CRC cell lines. Single cells clones of these cell lines were established which stably express a doxycycline inducible-CRISPR/Cas9 (44) designed to target the ILK gene. The cells were treated with 2 µg/ml doxycycline for 3 days, and ILK protein expression was examined in cell lysates by western blot after doxycycline withdrawal. The results (**Figure 9A**) showed that ILK protein expression in the CRC cell lines was inhibited by 80% to 90%. Therefore, the doxycycline inducible-CRISPR/Cas9 deleted the ILK gene sufficiently in the CRC cell lines to significantly reduce ILK protein expression allowing the effect on downstream signaling pathways to be tested. The CRC lines were screened by western blot for basal level PD-L1 protein expression which showed only RKO cells expressed PD-L1 (**Figure 9B**). Interestingly, ILK KD reduced the PD-L1 expression in RKO cells suggesting that maintaining ILK protein levels is necessary for basal PD-L1 expression in these cells (**Figure 9B**).

To determine the ILK-dependent mechanism involved in regulating basal PD-L1 expression in RKO cells, we determined PD-L1 expression in different conditions including basal level, starvation and serum restimulation after starvation at different time points. The results (**Supplementary Figure 5**) showed that ILK KD reduced PD-L1 expression independent of the conditions applied. Next, we examined the NF-κB p65 inflammatory signaling pathway since it positively regulates PD-L1 expression (56). The results showed that the NF-κB p65 showed a reduction in the total protein and that was reflected in the phosphorylation level of p65 (**Figure 9C**).

#### ILK KD Reduced IFNγ-Induced PD-L1 Expression in CRC Cell Lines

**Figure 10:**
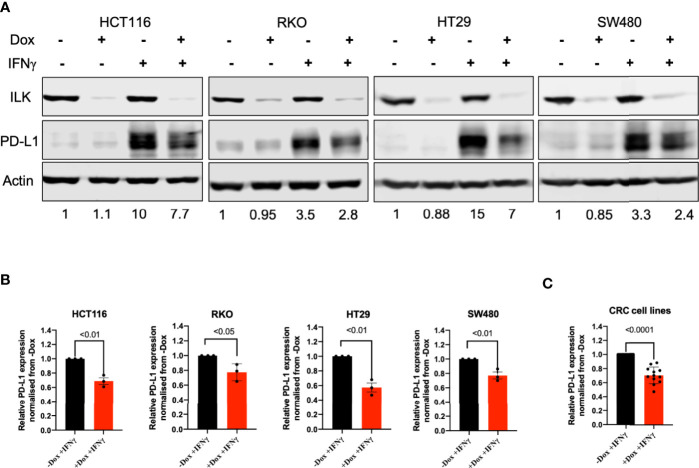
ILK KD reduced IFNγ-induced PD-L1 expression in the CRC cell lines. **(A)** Western blot showed PD-L1 induction by IFNγ (50 ng) for 24 hr in HCT116, RKO, HT29 and SW480 CRC cells. Actin was used as an internal control. The protein quantitation was determined by relative ratio to actin. The relative PD-L1 expression is normalized to the -Dox unstimulated protein. **(B)** The quantitation of PD-L1 expression in CRC cell lines. **(C)** The quantitation of PD-L1 protein expression in the different CRC cell lines combined. The relative PD-L1 expression is normalized to the -Dox protein. Error bars are represented as mean ± SEM (n = 3). *P*-value was analyzed with an unpaired t-test. The significant *P*-value is < 0.05.

As shown above except for RKO cells the other CRC cell lines did not express PD-L1 (**Figure 9B**). Since IFNγ is a strong inducer of PD-L1 (16, 53) we treated the different cell lines with this cytokine. The results (**Figure 10A**) showed that exposing the cells for 24 hr to IFNγ showed a clear induction of PD-L1. ILK KD in the CRC cell lines reduced the IFNγ-induced PD-L1 expression, most notably in the HT29 cells (**Figures 10A, B**). In addition, when the relative expression of PD-L1 in these different CRC cell lines is quantitated and blotted together and they displayed similar trends of PD-L1 expression (**Figure 10C**). This suggests that ILK is involved in PD-L1 regulation independent of the different genetic mutations carried by these cell lines.

#### ILK KD Reduced IFNγ-Induced PD-L1 Expression *via* Regulating NF-κB p65 in a Cell-Dependent Manner

**Figure 11:**
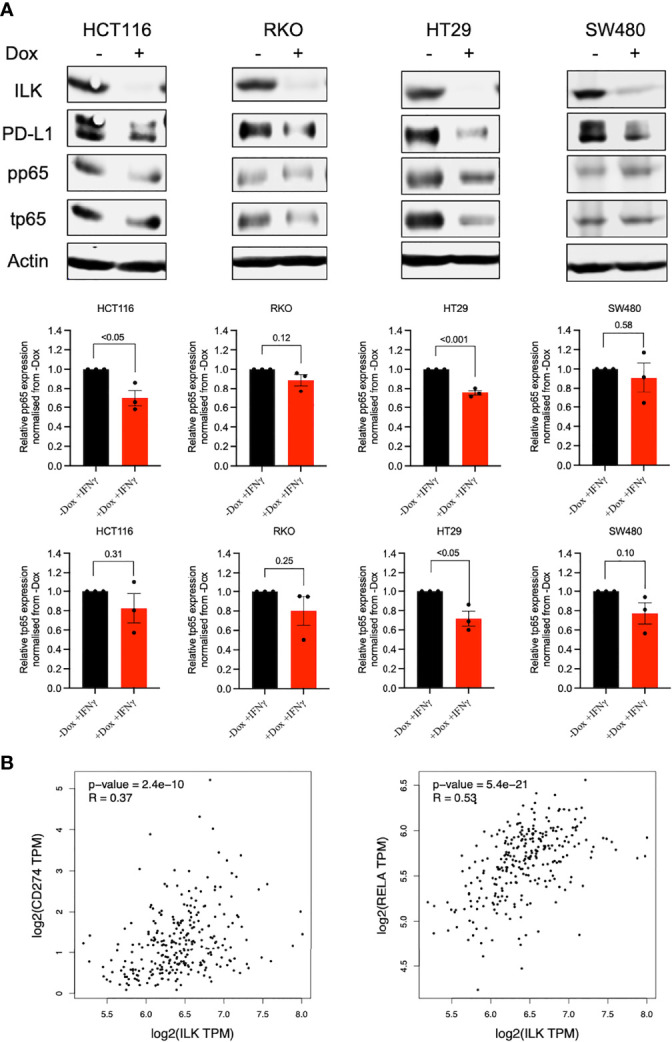
ILK KD reduced IFNγ-induced PD-L1 expression in the CRC cell lines in part *via* regulating NF-κB p65. [**(A)**, upper] western blot showing NF-κB p65 (total and phosphorylated) protein expression in CRC cell lines stimulated with IFNγ (50 ng) for 24 hr. [**(A)**, lower] the quantitation of NF-κB p65 (total and phosphorylated) protein expression. Quantitation of proteins expression normalized to -Dox. Actin was used as an internal control. Error bars are represented as mean ± SEM (n = 3). *P*-value was analyzed with an unpaired t-test. The significant *P*-value is < 0.05. [**(B)**, left] The correlation between ILK and CD274 gene expression in COAD tumors *via* GEPIA. [**(B)**, right] The correlation between ILK and Rela gene expression in COAD tumors *via* GEPIA. R is Spearman’s Rho values. Significant *P*-value is < 0.05.

Since ILK KD reduced PD-L1 basal expression was accompanied by reduced NF-κB p65 protein expression in RKO cells, we examined whether the CRC cell lines may also exhibit a similar phenotype following IFNγ stimulation. Accordingly, the CRC cells were treated with IFNγ for 24 hr and NF-κB p65 was examined by western blot. The results showed that while overall total and phosphorylated NF-κB p65 expression was reduced by ILK KD in the cell lines, only HT29 and HCT116 reached statistical significance (**Figure 11A**). HT29 cells showed a reduction in both total and phosphorylated NF-κB p65, while HCT116 cells displayed a reduction in the phosphorylation (**Figure 11A**). Overall, these results indicate that ILK dependent signaling pathways regulate PD-L1 expression in the CRC cell lines in a cell specific manner.

To confirm the above finding, the GEPIA platform was used to display the correlation among ILK, PD-L1 (CD274), and NF-κB p65, (Rela) gene expression in COAD tumors. The results showed that ILK expression has a significant positive correlation with CD274 (R=0.37 and *P*<0.0001) and Rela (R=0.53 and *P*<0.0001) gene expression (**Figure 11B**). This is consistent with the finding of protein expression in CRC cell lines. In addition, Rela and CD274 expression also displayed a positive correlation (R=0.30 and *P*<0.0001) in COAD (**Supplementary Figure 3**). Collectively, these results suggest an important role for ILK in CRC cell PD-L1 regulation at least in part *via* the NF-κB p65 signaling pathway.

#### ILK KD in CRC Cells Increased Sensitivity to NK92 Immune Cell Cytotoxicity

**Figure 12:**
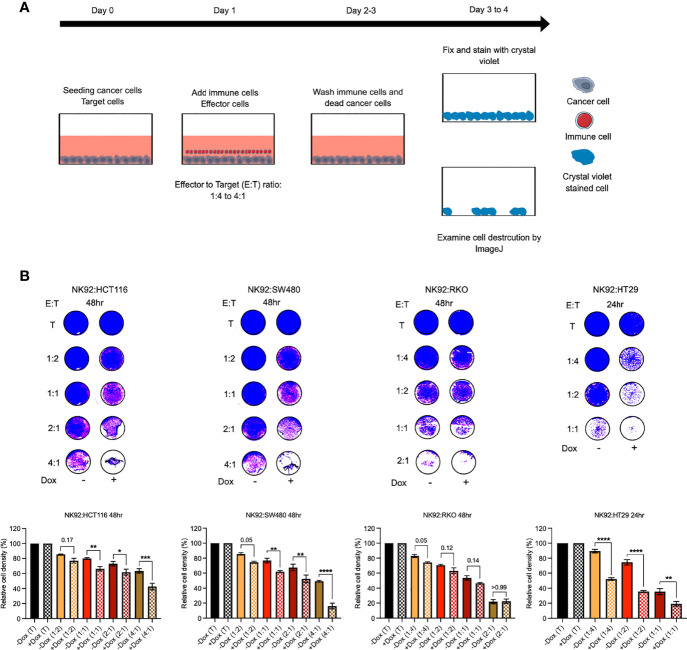
ILK KD in the epithelial CRC cells increased sensitivity to NK92 immune cell cytotoxicity. **(A)** The schematic illustration of co-culturing NK92 immune cells with CRC cells. At 0 time point the optimal -Dox and +Dox CRC cells number (target, T) were seeded into a 96-well plate. The following day after CRC cells adhesion, the immune cells (effector, E) were added over the target cells at different ratio (E:T). The immune cells were incubated for either 24hr or 48hr depending on CRC cell lines. Then, immune cells and any dead cancer cells were washed off by gently media change. The remaining adherent cells then were incubated for 24hr before fixing and staining. Following staining with crystal violet, the plates were imaged and cell densities and destruction by effector cells were quantitated by ImageJ software. **(B)** Killing activity of NK92 immune cells against the CRC cells. The control (T, target only) was replaced with only media without effector cells. The quantitation of cell density was normalized to control. Statistical significance (*P* < 0.05) was calculated by Ordinary One-way Anova with multiple comparisons. Error bars are represented as mean ± SEM (n = 3). *P-*value < 0.05 is significant. * (0.05) ** (< 0.01), ***(< 0.001) and **** (< 0.0001).

Since the above results showed a reduction in PD-L1 expression resulting from ILK KD we posited that this could sensitize CRC cells to immune cell cytotoxicity. To test this hypothesis, NK92 immune cells were co-cultured with CRC cells *in vitro* as described previously (57) (**Figure 12A**). HCT116, SW480 and RKO cells were co-cultured for 48hr whereas HT29 cells were co-cultured for 24hr. The results showed that NK92 cells displayed killing activity after 48hr against HCT116 and SW480 cells at 2:1 and higher ratios and against RKO cells at 1:2 and higher ratios (**Figure 12B**). In contrast, NK92 cells showed killing activity after only 24hr against HT29 cells at 1:2 ratio (**Figure 12B**). More importantly, ILK KD CRC cells were more sensitive to the NK92 cells compared with the control cells expressing ILK and this observation was more pronounced in HT29, SW480 and HCT116 respectively, but there was not a significant difference in RKO cells (**Figure 12B**). In addition, NK92 cells were able to kill a significant proportion of ILK KD HT29 cells at very low ratios, 1:4 and 1:2, compared with the control cells expressing ILK (**Figure 12B**). These results suggested that ILK expression in CRC cells increased resistance to NK92 cell cytotoxicity in a cell dependent manner but targeting ILK can overcome this.

## Discussion

Targeting the TME in cancer therapy is considered a challenge as there are complex interactions among different compartments for promoting cancer progression and immune evasion (11, 58, 59). To understand the functions and contributions of the TME in cancer progression it is essential to study and observe its multiple components. We have previously shown that myeloid-ILK deficiency reduced inflammation of experimental colitis by regulating neutrophil infiltration and cytokine production (32). While this suggested that ILK plays an important role in the inflammatory tumor microenvironment in colon cancer (36), mechanisms involved remain to be established.

Here we have demonstrated that ILK is upregulated in tumors and their adjacent non-tumor tissues in CRC and high expression of ILK is associated with poor prognosis and advanced stages that are associated with invasion and metastasis. While this is consistent with recently published observations (60), our analysis also showed that ILK expression is associated with different TME components including CAFs, immune cells, and cytokines/chemokines. We also observed that ILK is highly expressed in the TME because its expression in stromal and immune cells, in addition to the epithelial cancer cells in colon cancer. More importantly, we show a positive correlation between ILK expression and immunosuppressive cell infiltration as well as immune inhibitory molecules. Moreover, we demonstrate that ILK mediates PD-L1 protein expression in CRC cells and subsequently promotes a resistance to effector NK92 immune cells *in vitro* indicating a potential role in maintaining the immunosuppressive TME.

We have established here that ILK is not only upregulated in tumors but also in the surrounding tissues. This is consistent with what has been found in previous studies where tumor-adjacent tissues distant from tumors (up to 10 cm) showed dynamic abnormality ranging between healthy to tumor status (46, 61). This suggests that sampling sites could be a factor for contradictory differential expression results between the independent datasets or some immune cells infiltrated in the adjacent tissues that might have impact in gene expression. We showed that the primary tumors and their adjacent non-tumor tissues exhibit high expression not only of ILK but also immune and CAF gene signatures (**Supplementary Figure 4**). Taken together, ILK expression is upregulated in tumors and their adjacent non-tumor tissues supporting a role for ILK in regulating the TME and surrounding tissues.

To provide further evidence for such a role we evaluated different cellular components in TME *via* web-based mining databases. ILK has already been implicated in fibroblast and CAF regulation (4, 49) and in accord with this we found that ILK is significantly associated with CAF infiltration in different cancers with a notable correlation in COAD, STAD and LUSC. Moreover, CAFs are involved in EMT induction to facilitate invasion and metastasis (50), and ILK has been shown to induce EMT in colon cancer (62). Our results are consistent with these studies as we found that ILK is significantly correlated with advanced Duke’s stages of colon cancer which are accompanied by invasion and metastasis (63) and ILK expression is also correlated with EMT gene marker expression. Furthermore, we showed that high expression of ILK in COAD, STAD and LUSC was associated with a poorer survival rate. These findings suggest that ILK is a potential marker for poor prognosis, and it could be an important driver for regulating CAFs and EMT which potentially in turn promotes invasion and metastasis (50).

The infiltration of the immune cells in TME is influenced by cancer cells and CAFs which together are implicated in immune evasion (5, 64). Since our lab has shown that ILK expression in myeloid cells promotes neutrophil infiltration in experimental mice colitis (32), we posited that ILK might be involved in immune infiltration in the TME. We found that ILK expression is positively correlated with immune cell infiltration including CD4^+^ T cells, macrophages, neutrophils and DCs in COAD, LUSC and STAD. ILK expression also showed a negative correlation with tumor purity indicating that it is highly expressed in TME (38) possibly by different non-cancer cells. That is confirmed in cells from colon cancer *via* immune cell atlas platform as found that ILK is expressed in stromal cells, myeloid cells and TNKILC, and ILK expression in these cell types is higher than its expression in epithelial cancer cells. This suggests that the ILK negative correlation with tumor purity as well as ILK positive correlation with CAFs and immune cell infiltration is due to higher ILK expression in the TME non-cancer cells. This is also agreed with a previous study by Liu et al. that demonstrated ILK expression has a role in T-cell survival and chemotaxis (65), also this agreed with the other studies presenting a role for ILK in myeloid cells and fibroblasts (32, 49). These results suggest that non-cancer cells expressing ILK may have differential contributions to the TME.

Although there was a correlation between ILK and immune cell infiltration, that does not mean ILK is implicated in anti-tumor immunity. After investigating immunosuppressive cell infiltration, we found a correlation between ILK and immunosuppressive cell infiltration including Tregs and M2 macrophages. This is also confirmed and consistent with their gene markers. We noticed also that ILK expression is correlated with markers of TAM and M2 but not M1 macrophages in COAD, LUSC and STAD. TAM or M2 macrophage polarization are known to promote CRC progression *via* invasion, angiogenesis and metastasis (12, 66, 67). Previous studies found that ILK is implicated in macrophage polarization in an inflammation context (68, 69). However, in cancer context, this has not been investigated but our results indicate a possible function for ILK in macrophage polarization and infiltration in TME. Furthermore, ILK expression is correlated with cell infiltration and gene marker of Tregs in COAD, LUSC and STAD. Tregs plays an important role in TME by suppressing anti-tumor immunity (70, 71). In contrast, ILK does not show an association with immunosuppressive Treg and M2 cell infiltration, and their markers in KIRC suggesting the implication of ILK is dependent on tumor type.

An earlier study has established that ILK is important for DC polarization in response to integrin ligands which mediate migration and adhesion (30). DCs have been found to be affected in cancers that exhibit an antigen presentation dysfunction and immunosuppression (72, 73). In our study, DC infiltration and their gene markers were associated with ILK expression in 28 cancer types and significantly with COAD, STAD and LUSC, but showed a weaker correlation in KIRC. DCs are known to promote cancer progression in colon and other cancers by regulating and interacting with Tregs inducing T cell exhaustion (6, 9, 74–76). We show that ILK expression is significantly associated Treg infiltration as well as CTLA-4 and HAVCR2 (TIM-3) for T cell exhaustion (**Table 3**). Taken together, these results suggest that ILK impacts immunosuppression through regulating DC and T cell function and may be an important intracellular molecule regulating infiltrating immune cells and their functions in the TME.

ILK may also have a function in immune checkpoints regulating immune evasion and the immunosuppressive TME. We found that there was a pronounced correlation of ILK expression with most of common immune checkpoints ligands in COAD compared with the other cancers. These included PD-L1, PD-L2, Galectin-1, CD80, CD86, B7-H3 C10orf54, ADORA2A, IDO1 and Galectin-9. ILK expression is not only correlated with the above ligands but also with their receptors including PDCD1 (PD-1) and CTLA-4. Moreover, the results showed a strong correlation between ILK and Galectin-1, a gene that may be important for the exclusion of T cells from the TME as described recently (51). Also, Galectin-1 expression in lymphocytes reduces their proliferative and cytotoxic function (77). In addition, targeting Galectin-1 reduces infiltration of MDSCs and Treg cells, and increases CD4^+^ and CD8^+^ T cells (8). Galectin-1 knockdown also reduces macrophage polarization shift from M1 to M2 during glioblastoma progression in mice (8). While earlier studies have shown that PD-L1 and CTLA-4 blockade have therapeutic benefit in some cancers including melanoma and Hodgkins diseases, other cancers such as CRC except for a small subset of tumors are non-responsive (78). Moreover, the importance of PD-L1 expression in the TME may depend on cell type. Recently, Liu et, al found a significant association between PD-L1 expression in macrophage (but not in the tumor cells) with overall survival in non-small cell lung cancer (79). It remains possible that ILK is driving the expression of PD-L1 in these cases. Consequently, targeting ILK combined with immunotherapy could provide a new avenue for treating unresponsive solid tumors by suppressing co-inhibitory molecules.

Chemokine and cytokine secretion are important in recruiting and regulating immune cells as well as cancer progression (3, 10, 13). The ILK has been identified as involved in a deferential expression of CXC chemokines (31). We found ILK expression to be associated with the expression of several important chemokine and cytokine genes in COAD, STAD and LUSC, but not in KIRC. ILK has previously been shown to regulate CCL2 in HCT116 CRC cells (35). CCL2 expression mediated by snail positive tumor cells undergoing EMT is responsible for tumor progression involving tumor growth and metastasis, and immunosuppression (increase in PD-L1 expression) (80). Our findings uncovered a positive correlation between ILK and snail gene expression (SNAI1) as well as CCL2 and PD-L1. There are also limited studies showing an association between ILK and CCL8, CCL13, CCL21 and others. CCL21 is involved in chemoresistance and stem-like features in CRC *via* snail (13). Moreover, CCL8 has been demonstrated to recruit TAMs in cervical cancer (81).

IL-10 has been found to induce TAMs (M2 polarization) in colorectal cancer promoting cancer proliferation and invasion (82). Moreover, IL-17B expressed primarily in the stroma of CRC induced IL-6 and IL-8 (83, 84). Our results show a significant correlation with IL-10 and M2 infiltration. Also, there is a positive correlation with IL-17B, IL-6 and IL-8. Taken together, these results suggest that ILK could control the secretion of a wide range of chemokines and cytokines licensing their complex interactions.

The above discussed findings indicated that ILK is correlated with immunosuppressive TME factors but that seemed to be tumor specific. This might be *via* particular gene secretions in the TME such as CCL13, IL10 and other genes. For instance, CCL13 and IL10 gene expressions are implicated in Tregs and M2 macrophages infiltration in COAD, KIRC, LUSC and STAD. ILK expression in KIRC exhibited negative correlation with CCL13 and IL10 expression, which might be the result of not showing an ILK correlation with Treg and M2 infiltration. In COAD, LUSC and STAD, on the other hand, ILK expression showed significantly positive correlation with CCL13 and IL10 which possibly the result of displaying an ILK association with Tregs and M2 infiltration. Therefore, the ILK correlation with specific cytokine or chemokine gene expression might be implicated in predicting immune cell infiltration such as immunosuppressive Treg and M2 cells.

Although the discussion above is focused on non-cancer cells, and our overall observations indicate ILK expression in immune cells and CAFs as important factors in the TME, it has been established that ILK is overexpressed in the epithelial CRC cancer cells (23) and also our observation of ILK expression in epithelial colon cancer cells *via* immune cell atlas is consistent with the previous study. It is suggested that the overall contribution of ILK to the TME or cancer cells may depend on the presence or absence of specific type of immune cells or CAFs but this remains to be resolved. Nonetheless, the epithelial cancer cell is still very important cell type to be investigated because it is the origin of colon cancer and an important player in inducting TME modification.

While a previous study found that HLA-DR mediated signaling increased activation and expression of several signaling proteins including ILK and PD-L1 in melanoma cells (85), no studies have investigated a role for ILK in regulating PD-L1 protein expression in the epithelial cancer cells, particularly CRC. We have shown that ILK KD reduced PD-L1 protein expression not only at the basal level but also reduced the expression of IFNγ-induced PD-L1 in different cell lines. The ILK dependent regulation of PD-L1 expression is at least partially *via* NF-κB p65. Moreover, these *in vitro* observations are in line with the web mining database results showing a correlation of ILK gene expression with both CD274 (PD-L1) and Rela (NF-κB p65) in COAD. This is consistent with a previous study found that NF-κB regulates PD-L1 expression in gastric cancer (56). While we have linked ILK to PD-L1 expression *via* its IFNγ and NF-κB signaling, it remains to be determined whether this is a direct or indirect mechanism.

Since PD-L1 expression is involved in suppressing anti-tumor immune cell activity including NK cells (57, 86, 87), we examined whether ILK KD-mediated PD-L1 reduction in the CRC cells will display an effect in immune cell cytotoxicity. We found that ILK KD in the epithelial CRC cells enhanced the NK92 cell cytotoxicity, and this is in line with the reduction in PD-L1 expression. Moreover, the different CRC cell lines reacted differently toward NK92 cells likely the result of harboring different genetic mutations. For example, the cell lines HCT116 and SW480 harboring KRAS mutation (55),, showed more resistance and they required more time as well as a higher effector cell ratio to be killed. This is accord with a previous study that showed that the KRAS mutation in CRC drives immune response suppression and immunotherapy resistance (88). In addition, the KRAS mutant cell lines may lose their regulatory loop between ILK and KRAS compared with WT cell lines as reported previously (89). This suggests that targeting ILK in KRAS mutant cancer cells might show less impact in sensitizing cancer cells to immune cell cytotoxicity (89).

HT29 cells harboring a TP53 mutation but WT KRAS (55), displayed more sensitivity to NK92 immune cells with ILK KD. HT29 KD cells were killed in a shorter time as well as at a lower effector ratio compared with KRAS mutant cells. In contrast, the RKO cell line is WT TP53 and KRAS (55), and targeting ILK in this cell line did not display sensitivity to NK92 cells. This likely because that this cell line expresses a high level of PD-L1 at the basal level, which is markedly different from the other cell lines (**Figure 9B**). Therefore, our results suggested that targeting ILK in the epithelial CRC cells enhanced immune cell cytotoxicity but this depended on the genetic mutation background of the cancer cells, with TP53 mutant cells potentially being more sensitive in absence of ILK to the effector immune NK cells.

Our overall results of enhancing immune cell cytotoxicity as an effect of ILK KD in the epithelial CRC cells suggests that targeting ILK may be promising for solid tumors and could show clinical benefit when combined with other approved therapies including chemotherapy, radiotherapy, and potentially immunotherapy. These results also provide insight into the importance of adhesion molecules in cancer cells and their targeting as cancer therapeutic strategy (90).

While we provide a new insight about a potential role of ILK in immune evasion and immunosuppressive TME in solid tumors, particularly CRC, there are limitations in this study. The use of databases and the interpretations of the findings need to be validated in preclinical models *in vivo* and in further cohorts of patient tumor tissues. Moreover, previous studies have shown that inflamed (hot) tumors display a better response to PD-L1 inhibitors. In CRC this is the case for MSI tumors (37, 53). The CRC cell lines utilized here consist of both MSI and MSS origins, and both types showed a similar trend of PD-L1 reduction enhancing immune cell killing activity as an effect of ILK KD regardless to their microsatellite status. However, the *in vitro* experiments do not reflect the *in vivo* TME of MSI tumors where there is a lack of different immune cell types infiltration (91). A further limitation of this study is evidence for secreted specific cytokines or chemokines by CRC cells which could predict specific immune cell infiltration, a potentially important area for future studies.

In summary, elevated ILK is associated with poor prognosis in COAD and is upregulated in the TME and in the adjacent non-tumor tissues. ILK is expressed in different cell types in colon cancer including epithelial cancer, stromal and immune cells. ILK possibly regulates immune cell functions including macrophage polarization and T cell exhaustion. Different critical immunosuppressive factors in the TME show positive association with ILK expression including CAFs, Tregs and M2 macrophages infiltration as well as PD-L1 expression in a tumor-specific tumor manner. ILK-dependent signaling regulates basal and induced PD-L1 expression in the epithelial CRC cell lines and promotes resistance of CRC cells to immune cell killing activity *in vitro.* Accordingly, this pseudokinase is likely an important component of the immunosuppressive TME and may potentially be involved in the counterbalance between the anti-tumor immune response and inhibitory factors promoting immune evasion. Targeting ILK in combination with immunotherapy could be promising for TME modulation and growth suppression of solid tumors.

## Data Availability Statement

The original contributions presented in the study are included in the article/**Supplementary Material**, further inquiries can be directed to the corresponding author/s.

## Author Contributions

SA, AA, and BW planned the study; SA performed the experiments; BW, AA, and RB reviewed the results; SA and BW wrote and AA and RB edited the manuscript. All authors contributed to the article and approved the submitted version.

## Funding

SA was supported by a post-graduate scholarship from Najran University, Najran, Saudi Arabia. Research at the Hudson Institute of Medical Research is supported Victorian Government’s Operational Infrastructure Support Program.

## Conflict of Interest

RB is Chief Scientific Officer and shareholder of Catherics Pty Ltd. BW is a Director and shareholder of this company.

The remaining authors declare that the research was conducted in the absence of any commercial or financial relationships that could be construed as a potential conflict of interest.

## Publisher’s Note

All claims expressed in this article are solely those of the authors and do not necessarily represent those of their affiliated organizations, or those of the publisher, the editors and the reviewers. Any product that may be evaluated in this article, or claim that may be made by its manufacturer, is not guaranteed or endorsed by the publisher.
