# Supplementary material for: Integrin-Linked Kinase Expression Characterizes the Immunosuppressive Tumor Microenvironment in Colorectal Cancer and Regulates PD-L1 Expression and Immune Cell Cytotoxicity
Source: Front Oncol. 2022 May 25;12:836005. doi: 10.3389/fonc.2022.836005 (PMC9174997; doi:10.3389/fonc.2022.836005)
Supplement: Supplementary file 1 [file DataSheet_1.docx]

**Supplementary Table 1.** Correlation between ILK expression and immune cell markers and related genes expression in COAD, KIRC, STAD and LUSC via TIMER.

CODA, colon adenocarcinoma; KIRC, kidney renal clear cell carcinoma; STAD, stomach adenocarcinoma; LUSC, lung squamous cell carcinoma; TAM, tumor-associated macrophage; Th, T helper cell; Tfh, Follicular helper T cell; Treg, regulatory T cell; Cor, Rho value of Spearman’s correlation; None, correlation without adjustment. Purity, correlation adjusted by purity. Significant *P*-value is <0.05 (bold); **P* < 0.01; ***P* < 0.001; ****P* < 0.0001.

**Supplementary Figure 1.** The correlation between ILK expression and survival rates in KIRC, STAD and LUSC. The LUSC patients were divided into low and high ILK mRNA expression groups, below and above the cut-off (2.16 FPKM) respectively. Low ILK expression patients (n=164) and high ILK expression patients (n=330). The STAD patients were divided into low (n=270) and high (n=84) ILK mRNA expression groups, below and above the cut-off (3.86 FPKM) respectively. The KIRC patients were divided into low (n=133 and high (n=395) ILK mRNA expression groups, below and above the cut-off (3.80 FPKM) respectively. KIRC, kidney renal clear cell carcinoma; STAD, stomach adenocarcinoma; LUSC, lung squamous cell carcinoma. Significant *P*-value is <0.05.


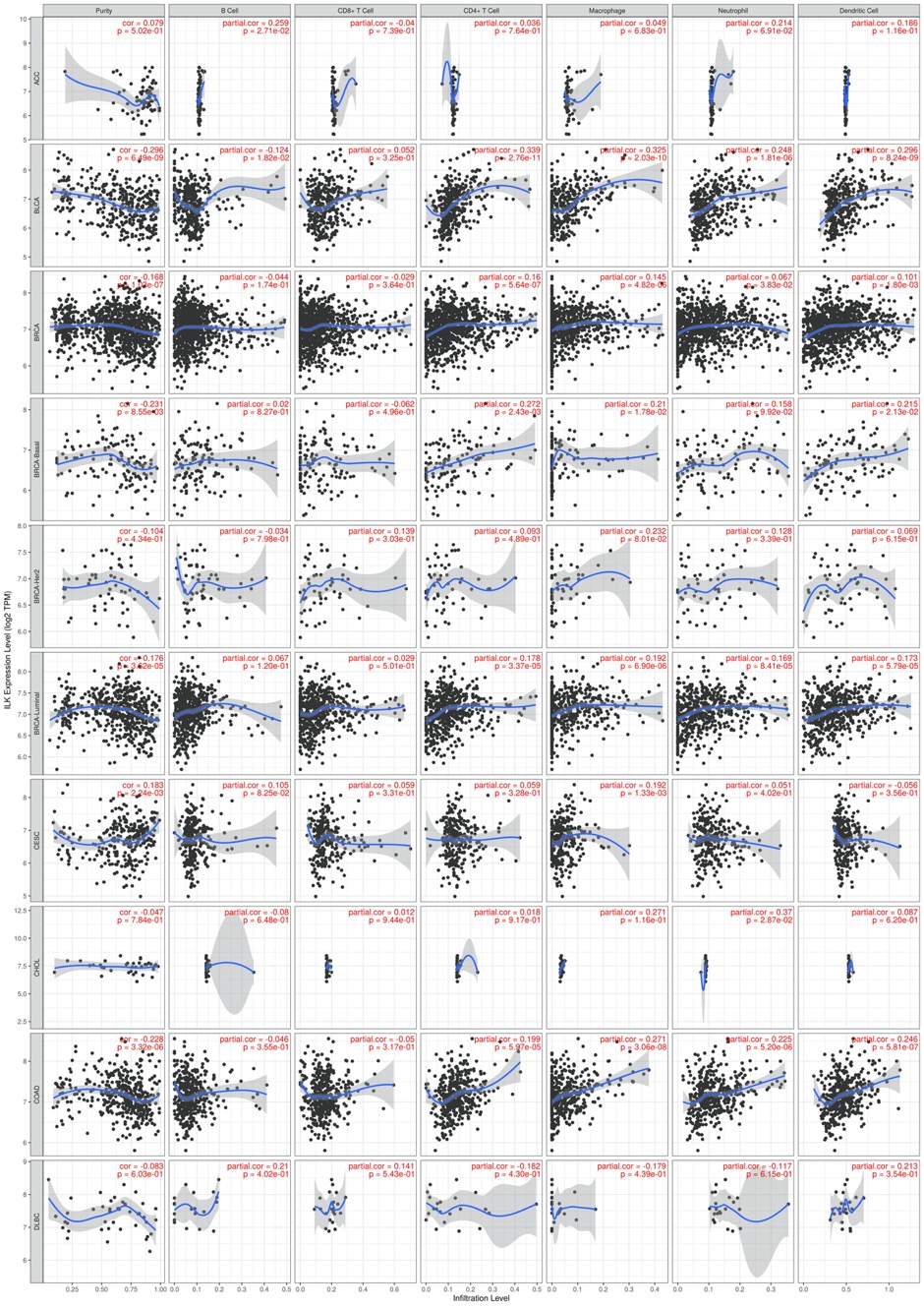


**Supplementary Figure 2.** Correlation between ILK expression and immune cells infiltration in 39 cancers. The correlations between ILK gene expression and tumor purity are on the left panel. The correlation between ILK and immune cells including B cells, CD8+ T cells, CD4+ T cells, macrophages, neutrophils and DCs are corrected by tumor purity (partial.cor, partial spearman’s correlation rho). Significant *P*-value is <0.05. Adrenocortical carcinoma(ACC), Bladder Urothelial Carcinoma (BLCA), Breast invasive carcinoma (BRCA), Breast invasive carcinoma-Basal (BRCA-Basal), Breast invasive carcinoma-Her2 (BRCA-Her2), Breast invasive carcinoma-Luminal (BRCA- Luminal), Cervical squamous cell carcinoma and endocervical adenocarcinoma(CESC), Cholangio carcinoma(CHOL), Lymphoid Neoplasm Diffuse Large B-cell Lymphoma(DLBC), Esophageal carcinoma(ESCA), Glioblastoma multiforme(GBM), Head and Neck squamous cell carcinoma(HNSC), Head and Neck squamous cell carcinoma- HPVpos(HNSC-HPVpos), Head and Neck squamous cell carcinoma-HPVneg(HNSC-HPVneg), Kidney Chromophobe(KICH), Kidney renal clear cell carcinoma(KIRC), Kidney renal papillary cell carcinoma(KIRP), Brain Lower Grade Glioma(LGG), Liver hepatocellular carcinoma(LIHC), Lung adenocarcinoma(LUAD), Mesothelioma(MESO), Ovarian serous cystadenocarcinoma(OV), Pancreatic adenocarcinoma(PAAD), Pheochromocytoma and Paraganglioma(PCPG), Prostate adenocarcinoma(PRAD), Rectum adenocarcinoma(READ), Sarcoma(SARC), Skin Cutaneous Melanoma(SKCM), Skin Cutaneous Melanoma-Primary(SKCM- Primary), Skin Cutaneous Melanoma-Metastasis(SKCM- Metastasis), Testicular Germ Cell Tumors(TGCT), Thyroid carcinoma(THCA), Thymoma(THYM), Uterine Corpus Endometrial Carcinoma(UCEC), Uterine Carcinosarcoma(UCS), Uveal Melanoma(UVM).

**Supplementary Figure 3.** The correlation between Rela and CD274 gene expression in COAD via GEPIA. R is Spearman’s correlation Rho value. Significant *P*-value is <0.05.

**Supplementary Figure 4.** Immune and CAF gene signatures in primary tumors and their adjacent non-tumor tissues compared with non-cancer tissues. The dataset GSE95132 was downloaded from GEO database. This dataset includes 10 CRC patients (10 CRC tumor tissues (Tumor) and their 10 adjacent non-tumor tissues (Adjacent)) and 5 non-cancer individuals (5 normal colonic crypts (Normal)). (A) The heat map displays gene expressions (Log2(TPM+1)) of all immune and CAFs gene signatures as well as ILK. (B) ILK and representative gene markers for M2 (CD163), Treg (FOXP3) and CAF (ACTA2). Error bars are represented as mean ± SEM. *P*-value was analyzed with an unpaired t-test. Significant *P*-value is <0.05. *P*-value was analyzed with an unpaired t-test. The significant *P*-value is <0.05; ns, not significant. (T) CRC tumor tissues; (N) their adjacent non-tumor tissues.

**Supplementary Figure 5.** The western blot showed the effect of ILK KD on PD-L1 expression in RKO cells at different conditions including basal level or non-starved (NS), overnight starvation (0 hr) and serum stimulation after overnight starvation at indicated time points (1 hr, 2 hr, 4 hr and 8 hr). Quantitation of protein expression normalized to -Dox (NS). Actin was used as an internal control.
